# Mapping the Interactome of the Nuclear Heparan Sulfate Proteoglycan Syndecan-1 in Mesothelioma Cells

**DOI:** 10.3390/biom10071034

**Published:** 2020-07-11

**Authors:** Ashish Kumar-Singh, Jatin Shrinet, Malgorzata Maria Parniewska, Jonas Fuxe, Katalin Dobra, Anders Hjerpe

**Affiliations:** 1 Division of Pathology, Department of Laboratory Medicine, Karolinska Institutet, SE-14186 Stockholm, Sweden; Ashish.Kumar.Singh@ki.se (A.K.-S.); jonas.fuxe@ki.se (J.F.); malgorzata.parniewska@ki.se (M.M.P.); anders.hjerpe@ki.se (A.H.); 2 Department of Biological Science, Florida State University, Tallahassee, FL 32306, USA; jatbioinfo@gmail.com; 3 Division of Clinical Pathology/Cytology, Karolinska University Laboratory, Karolinska University Hospital, SE-14186 Stockholm, Sweden

**Keywords:** Syndecan-1, nucleus, proteomics, immunoprecipitation, mesothelioma, exon junction complex

## Abstract

Syndecan-1 (SDC1) is a cell surface heparan sulfate proteoglycan (HSPG), which regulates various signaling pathways controlling the proliferation and migration of malignant mesothelioma and other types of cancer. We have previously shown that SDC1 can translocate to the nucleus in mesothelioma cells through a tubulin-dependent transport mechanism. However, the role of nuclear SDC1 is largely unknown. Here, we performed co-immunoprecipitation (Co-IP) of SDC1 in a mesothelioma cell line to identify SDC1 interacting proteins. The precipitates contained a large number of proteins, indicating the recovery of protein networks. Proteomic analysis with a focus on nuclear proteins revealed an association with pathways related to cell proliferation and RNA synthesis, splicing and transport. In support of this, the top RNA splicing candidates were verified to interact with SDC1 by Co-IP and subsequent Western blot analysis. Further loss- and gain-of-function experiments showed that SDC1 influences RNA levels in mesothelioma cells. The results identify a proteomic map of SDC1 nuclear interactors in a mesothelioma cell line and suggest a previously unknown role for SDC1 in RNA biogenesis. The results should serve as a fundament for further studies to discover the role of nuclear SDC1 in normal and cancer cells of different origin.

## 1. Introduction

The syndecan (SDC) family consists of four structurally conserved type I transmembrane proteins that are found on the cell surface. SDC1, which is also known as CD138, is the major syndecan in epithelial cells [1]. This type of heparan sulfate proteoglycans (HSPG) contain an extracellular domain with glycosaminoglycan (GAG) side chains, a transmembrane domain and a short cytoplasmic domain [2]. Their cytoplasmic regions are involved in interactions with cytoskeletal proteins, thereby influencing the dynamics of the actin cytoskeleton and membrane trafficking. All these interactions help in optimizing syndecan recycling via endosomal compartment, also facilitating the internalization of accompanying protein cargo, as well as regulating cell adhesion and other signaling systems [3,4].

The SDCs are involved in many different biological processes, such as cell differentiation, adhesion, spreading and migration [5,6]. Traditionally the role of SDC1 has been seen mainly as taking place exclusively at the cell membrane. However, we and others have discovered that SDC1 can also translocate to the nucleus in mesothelioma cells [7], as well as in other cells [7,8,9,10]. Simultaneous presence in the nucleus of both the cytoplasmic and extracellular domains, including the heparan sulfate side chains, indicates the translocation of the entire SDC1 molecule [10]. This nuclear translocation of SDC1 is tubulin-dependent [7], and the juxtamembrane sequence motif RMKKK acts as a nuclear localization signal [10,11,12,13].

The nuclear translocation of SDC1 hampers cell proliferation [14]. In the cell membrane, SDC1 binds to different growth factors and their related receptors to form a complex and plays role in the regulated co-translocation of SDC1 and FGF-2. Both SDC1 and FGF-2 can be found co-localized in the nucleus. SDC1 overexpression leads to nuclear accumulation of FGF-2, which confirms the functional role of SDC1 in the nuclear transport [10]. Heparanase, an endoglycosidase that cleaves HS and substantially reduces the nuclear SDC1 levels in a concentration-dependent manner, is an important factor in regulating the levels of HS and SDC1 in the nucleus [8].

The more precise interactions of SDC1 in the nucleus, however, still remain to be clarified. To gain further insight into the function of nuclear SDC1, we have identified nuclear proteins binding to SDC1 using co-immunoprecipitation and proteomic analysis.

## 2. Materials and Methods

### 2.1. Cell Lines and Cell Culture Conditions

The study was performed using the STAV-AB mesothelioma cell line in which the nuclear translocation of SDC1 was first demonstrated. SDC1 shows distinct nuclear translocation in these cells at 48 h after seeding [7]. This cell line shows epithelioid morphology when grown in RPMI 1640 medium, containing 25 mM HEPES (GIBCO, Grand Island, NY, USA) and 2 mM L-Glutamine, supplemented with 10% human AB serum. The effects of SDC1 expression were studied after stable transfection with full length SDC1, compared to empty vector control [13,14] and after SDC1 silencing with siRNA. The silencing of the SDC1 expression was done by seeding the STAV-AB cells and, after one day, transfecting them using Lipofectamine^TM^ 2000 (Invitrogen, Carlsbad, CA, USA). A pool of three siRNA constructs specific for SDC1 were used (Ambion^®^, Inc., Stockholm, Sweden) with an optimized concentration of 40 nM [14]. Scrambled siRNA was used as a control. The effects of transfection and silencing on the cellular content of SDC1 were always controlled by FACS analysis of each cell harvest.

### 2.2. Fluorescence Activated Cell Sorting (FACS)

SDC1-transfected cells were obtained from a previous study [12]. Retained SDC1 expression was verified by FACS analysis prior to the experiments. Cells were detached with enzyme-free Cell Dissociation Buffer (Gibco, 13,151–014) for 15 min and, when necessary, scraped off the plastic surface. Following fixation in 2% buffered formaldehyde for 10 min at 37 °C, the cells were incubated with antibodies against SDC1 (CD138) (1:20 dilution, Catalog no. MCA2459; Bio-Rad, Stockholm, Sweden) for 15 min at 4 °C and Alexa 488-conjugated goat anti-mouse secondary antibody (Catalog no. A-110017; Invitrogen, Stockholm, Sweden) for 15 min at room temperature in the dark. Subsequent experiments were performed when SDC1 showed more than a 1.5-fold increase in STAV-AB full length SDC1 cells compared to control cells.

### 2.3. Immunoprecipitation and Western Blot Analysis

For immunoprecipitation, sub-confluent STAV-AB cells were lysed using IP lysis buffer (Catalog no. 87788; ThermoFisher Scientific, Uppsala, Sweden) with protease inhibitor (Catalog no. 87786; ThermoFisher Scientific, Uppsala, Sweden). The protein concentration was determined by the Pierce BCA Protein Assay (Catalog no. 23228/23224; Thermoscientific, Waltham, MA, USA), using bovine serum albumin (BSA) as a standard. Initially, cell lysates containing 260 µg protein were incubated for 1 h at 4 °C with 0.5 mL Dynabeads Protein A and 0.5 mL Dynabeads Protein G (Catalog no. 10001D and 10003D; Invitrogen, Gothenburg, Sweden). The supernatants were collected followed by incubation overnight at 4 °C with corresponding Dynabead Protein A/G mixtures, which had been pre-incubated with polyclonal rabbit anti-SDC1 (Catalog no. 36-2900; Invitrogen, Gothenburg, Sweden; 100 µg per mL of mixture) and, as negative controls, beads were incubated with mouse IgG1 (Catalog no. X0931, Dako, CA, USA). After stringent washes, the immunoprecipitated proteins were released from the beads by boiling at 95-100 °C for 5 min. For verification, a similar immunoprecipitate was prepared using a different SDC1 antibody (Catalog no. MA5-12400; Invitrogen, Gothenburg, Sweden).

The presence of SDC1, EWSR1 and FUS in the immunoprecipitates obtained using the two different SDC1 antibodies were verified by Western blotting, using SDS polyacrylamide gel electrophoresis and transferred to a PVDF membrane using the trans-blot turbo transfer system (Bio-Rad), as shown in Figure S1. The membranes were blocked with 5% milk in PBS and incubated overnight at 4 °C with primary antibodies against SDC1 (2 µg/mL, rabbit anti-SDC1; Invitrogen, Sweden), EWSR1 (1:1000 rat monoclonal anti-EWSR1/EWS, ab252829, Abcam, Cambridge, UK) or FUS (1:500, mouse monoclonal anti-TLS/FUS, ab154141, Abcam, Cambridge, UK). Following washes, the membranes were developed with secondary antibodies, ECL Anti-rabbit IgG, Horseradish peroxidase-linked species-specific F(ab’)2 fragment (1:5000 dilution; GE Healthcare, Uppsala, Sweden) and chemiluminescent HRP Substrate (Advansta, Catalog no. K-12043-D10). The Odyssey Imaging System (LI-COR) was used to quantify the relative expressions of SDC1.

### 2.4. Proteomic Analysis

The proteomic screening was performed at the Karolinska Bioinformatic Center. Precipitated proteins were reduced in 1 mM DTT at room temperature for 30 min, followed by alkylation in the dark at room temperature, using 5 mM iodoacetamide for 20 min. Remaining iodoacetamide was quenched by the addition of 5 mM DTT. Digestion was carried out by the addition of 1 µg Trypsin (Pierce) and over-night incubation at 37 °C. The supernatant was then collected and cleaned by a modified sp3 protocol [15]. Briefly, 20 µL Sera-Mag SP3 bead mix (10 µg/µL) was added, followed by acetonitrile to achieve a final concentration of >95%. After 8 min incubation at room temperature, the beads were recovered using a magnetic rack. Following washes, the samples were removed from the magnetic rack. This procedure was repeated once, and after the samples were transferred to an MS-vial containing 2 µL of 10% formic acid in water.

Q-Exactive Online liquid chromatography–mass spectrometry (LC–MS) was performed using a Dionex Ultimate™ 3000 RSLCnano System coupled to a Q-Exactive mass spectrometer (Thermo Scientific). First, 5 µL was injected from each sample. Samples were trapped on a C18 guard desalting column (Acclaim PepMap 100, 75 µm × 2 cm, nanoViper, C18, 5 µm, 100 Å), and separated on a 50 cm long C18 column (Easy spray PepMap RSLC, C18, 2 µm, 100 Å, 75 µm × 15 cm). The nano capillary solvent A was 95% water, 5% DMSO and 0.1% formic acid. Solvent B was 5% water, 5% DMSO, 95% acetonitrile and 0.1% formic acid. At a constant flow of 0.25 μL min^−1^, the curved gradient went from 6% B up to 43% B in 180 min, followed by a steep increase to 100% B in 5 min.

FTMS master scans with 60,000 resolution (and mass range 300–1500 m/z) were followed by data-dependent MS/MS (30,000 resolution) on the top five ions using higher energy collision dissociation (HCD) at 30% normalized collision energy. Precursors were isolated with a 2 m/z window. Automatic gain control (AGC) targets were 1 × 10^6^ for MS1 and 1 × 10^5^ for MS2. Maximum injection times were 100 ms for MS1 and MS2. The entire duty cycle lasted ~2.5 s. Dynamic exclusion was used with 60 s duration. Precursors with unassigned charge state or charge state 1 were excluded. An underfill ratio of 1% was used.

We used a precursor ion mass tolerance of 10 ppm and product ion mass tolerances of 0.02 Da for HCD-FTMS and 0.8 Da for CID-ITMS. The algorithm considered tryptic peptides with a maximum of two missed cleavages—carbamidomethylation (C) as fixed modification and oxidation (M) as a variable modification.

### 2.5. Immunofluorescense

STAV-AB cells were seeded on cover slips in 24-well plates and fixed, after 24 h, in 4% PFA for 15 min at room temperature. Cells were permeabilized using 0.1% Triton in PBS (PBST) for 15 min. Blocking was performed for 1 h with 3% Bovine Serum Albumin (BSA) in PBST. Co-staining was then performed with a combination of antibodies against SDC1 (1:500, rabbit monoclonal anti-SDC1, ab128936, Abcam, Cambridge, UK) and either EWSR1 (1:200, rat monoclonal anti-EWSR1/EWS, ab252829, Abcam) or FUS (1:200, mouse monoclonal anti-TLS/FUS, ab154141, Abcam, Cambridge, UK) in 0.1% BSA in PBST. The following secondary antibodies were used: goat anti-rabbit 555 for SDC1, goat anti-rat 488 for EWSR1 and goat anti-mouse 488 for FUS detection (ThermoFisher, Stockholm, Sweden). Cells were mounted and imaged through a Zeiss LSM800 confocal microscope (Stockholm, Sweden). Images were imported into Image J.

### 2.6. Bioinformatic Analysis

The workflow of the analysis is shown in supplementary Figure S2. Both the negative control and the positive control libraries were run in triplicates. The peptides in each library were identified using Proteome Discoverer 1.4 (Thermo Scientific) against the human protein database and filtered to a 1% false discovery rate (FDR) cut-off. The respective replicate libraries were merged for further analysis. The proteins common between the negative and positive control libraries were removed and the remaining proteins were subjected to subcellular localization and functional annotation.

#### 2.6.1. Subcellular Localization and Functional Annotation

The subcellular localization of the proteins was performed by extracting information from the Human Protein Atlas database (https://www.proteinatlas.org) [16,17,18,19,20,21,22], SubCell Barcode database (http://subcellbarcode.org/) [23] and using a web tool—DeepLoc-1.0: Eukaryotic protein subcellular localization predictor (http://www.cbs.dtu.dk/services/DeepLoc/) [24]. SubCell Barcode provides the subcellular localization information for proteins of 12,418 genes from five different cell lines. DeepLoc-1.0 was trained on experimentally validated Uniprot proteins and uses Neural Network algorithms to predict the subcellular localization of the provided proteins. The proteins were classified into their respective pathways using the Webgestalt [25]. The testing of multiple gene sets and the adjustment of *p*-values were done using the Benjamini–Hochberg test. The pathways with *p*-value < 0.05 were considered to be significant.

#### 2.6.2. Protein Interaction Network

The protein–protein interaction (PPI) network of humans was downloaded from the STRING database (https://string-db.org) [26]. The SDC1-interacting proteins were mapped on to the PPI network and a sub network was extracted from the meta network, as shown in Figure S3. The proteins in the network were clustered according to their subcellular localization. The network was visualized using Cytoscape (https://cytoscape.org) [27].

### 2.7. RT-qPCR Analysis

SDC1 interactions with proteins associated with RNA transcription and export were indicated using proteomic analysis. We, therefore, measured how SDC1 expression influenced the total amounts of RNA and RNA coding for the housekeeping genes, actin and GAPDH. For this purpose, RNA was extracted using the High Pure Isolation Kit (Roche, Mannheim, Germany). Recovered cells were suspended in 200 µL PBS on ice, followed by 400 µL lysis buffer. The sample was applied to the upper reservoir of the high pure filter tubes and centrifuged for 15 s at 10,000 g to remove the low molecular compounds. DNA was then degraded by adding 10 µL DNase I and 90 µL DNase incubation buffer and removed by centrifugation after 15 min incubation at 15–25 °C. The RNA was recovered after the addition of 100 µL elution buffer and centrifugation at 10,000 g for 1 min. The total lysate contents of DNA and of isolated RNA were determined with the nanodrop technique.

The First Strand cDNA synthesis Kit (Catalog no. 27-9261-01; GE Healthcare, Uppsala, Sweden) was used to prepare cDNA, starting with 2 μg RNA. Briefly, denatured RNA was obtained by heating to 65 °C for 10 min, and then chilling on ice. Then, 5 μL of the Bulk First-Strand cDNA reaction mix was added together with 1 μL DTT solution and 1 μL pd (N_6_) primer to the heat denatured RNA. This reaction mix was then incubated at 37 °C for 1 h.

The subsequent qPCR was performed with the Platinum^®^ SybrGreen qPCR SuperMix-UDG Kit (Invitrogen^®^) using DNA-polymerase and a set of sense/antisense primers (CyberGene AB, Solna, Sweden). The primers had been designed using gene sequences of target genes obtained from GeneBank (NCBI) [12].

All reactions were performed in triplicate using an iCycler (CFX96^TM^ Real Time PCR Detection System, BioRAD Hercules, CA, USA). Analysis was done with Bio-Rad CFX Manager Software 2.0 (BioRad Laboratories 2008) and the amount of specific amplimer was related to the original sample content of DNA, determining the ratios between treated cells and their respective controls.

### 2.8. Statistical Analysis

All the statistical analyses were performed using GraphPad prism (version 6.01) software. Differences were evaluated using Student’s *t*-test and one-way completely randomized variance analysis (ANOVA). The plots were generated using GraphPad prism (version 6.01) software. *p*-value and FDR ≤ 0.05 were considered significant in all the analyses.

## 3. Results

### 3.1. Identification of SDC1-Interacting Proteins

**Figure 1:**
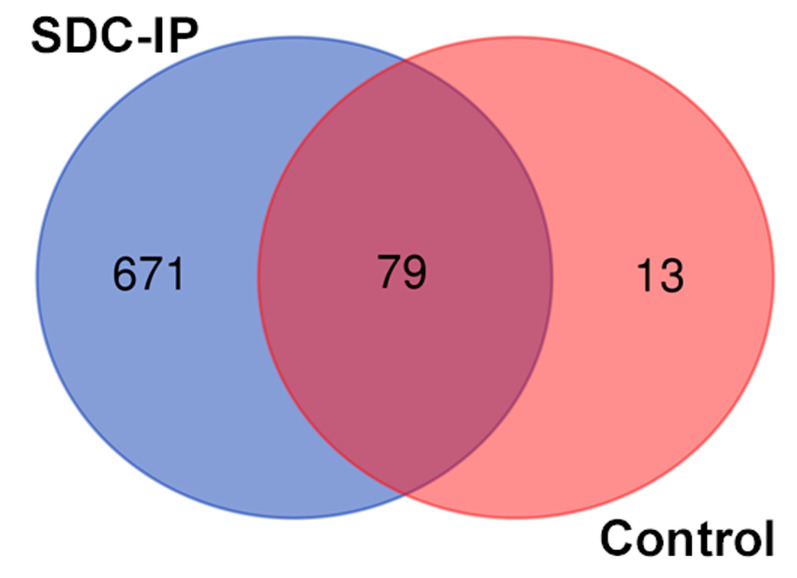
Venn diagram representing overlap between SDC1 co-precipitating proteins and control sample (precipitate obtained with mouse IgG but without the addition of the CD138 antibody).

After the Co-IP of the SDC1, LC/MS was performed on negative control and SDC1-specific samples (in triplicates) to identify the interacting partners of the SDC1 protein by searching the profiles against the uniprot database. A total of 750 proteins were identified in the precipitate using the SDC1-specific antibody, while 92 were found in the non-specific controls, as shown in Figure 1 and Table S1. A total of 79 proteins were common between the two samples and were removed from further analysis, leaving 671 proteins for downstream analysis. Tubulin, which is involved in the nuclear translocation of SDC1 [7], was present in the immunoprecipitated, supporting the correct design of the study.

### 3.2. Sub-Cellular Localization of Interacting Proteins and Related Pathways

**Figure 2:**
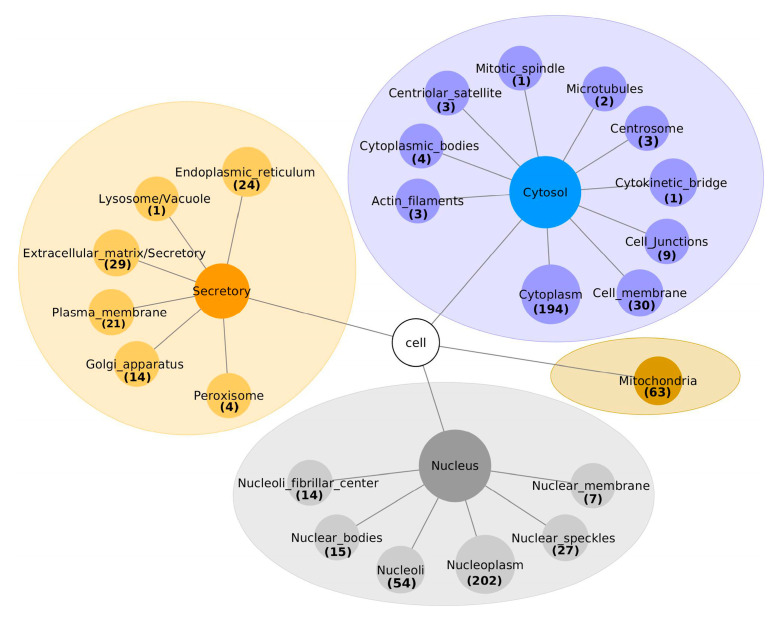
The sub-cellular localization of proteins interacting with SDC1, classified into cytosol, mitochondria, nucleus and extracellular matrices.

Two approaches were used to assign the sub-cellular locations to the 671 interacting proteins. Firstly, already known sub-cellular locations were extracted, using the Human Protein Atlas database and SubCell Barcode database. Secondly, the DeepLoc-1.0 was used for predicting the sub-cellular localization of the remaining proteins. Including proteins assigned to multiple locations, 314/671 (46.8%) were associated with the nucleus, as shown in Figure 2 and Table S1. Of these 314 nuclear proteins, 202 (64.3%) were localized in the nucleoplasm, 54 (17%) in the nucleoli, 27 (8.6%) in nuclear speckles, 15 (4.8%) in nuclear bodies, 7 (2%) in the nuclear membrane and 14 (4.5%) in the nucleoli fibrillar center. Interestingly, the Deeploc algorithm classified SDC1 as a cellular membrane protein, while the SubCell Barcode database categorizes it as a secretory protein.

The SDC1-interacting proteins were further classified into different pathways using Webgestalt web server, as shown in Figure S4. The analyses for this pathway were performed in two steps: (i) pathway analysis of all the interacting proteins; (ii) pathway analysis of nuclear proteins. The analysis of all interacting proteins predicted a total of 28 over-represented pathways, while the second analysis of nuclear proteins predicted eight, as shown in Table 1. Interestingly, the top three significant pathways, namely Spliceosome, Ribosome and RNA transport, were found in both analyses.

When the analysis focused on nuclear proteins, additional pathways of interest were indicated—mRNA surveillance pathway, Non-homologous end-joining and Cell cycle. Proteins related to Herpes simplex infection and Oocyte meiosis were also found, although they were considered to be of less interest in this context. The exon junction complex (EJC) seems to play an important role in these pathways, linking them together, as shown in Figure S5. The proteins, apoptotic chromatin condensation inducer 1 (ACIN1), eukaryotic translation initiation factor 4A3 (eIF4AIII), mago homolog (MAGOH), exon junction complex subunit and RNA binding motif protein 8A (Y14) were found to be common among the Spliceosome, RNA transport and mRNA surveillance pathways. Similarly, three proteins—RNA binding protein with serine rich domain 1 (RNPS1), Serine and argentine repetitive matrix 1 (SRRM1/SRm160) and Regulator of nonsense transcripts 1 (Upf1), RNA helicase and ATPase were common between the RNA transport and mRNA surveillance pathway.

Other proteins of interest, namely EDC3, EDC4 (enhancer of mRNA-decapping proteins), Ewing sarcoma RNA Binding Protein (EWSR1), Serine and Arginine Rich Splicing Factor 3 (SRSF3) and RNA-binding protein (FUS), were also identified as interactors of SDC1. Out of these, EWSR1 and FUS were selected on the basis of higher average peak area values and validated using Western blotting and immunofluorescence, as shown in Table 2.

**Figure 3:**
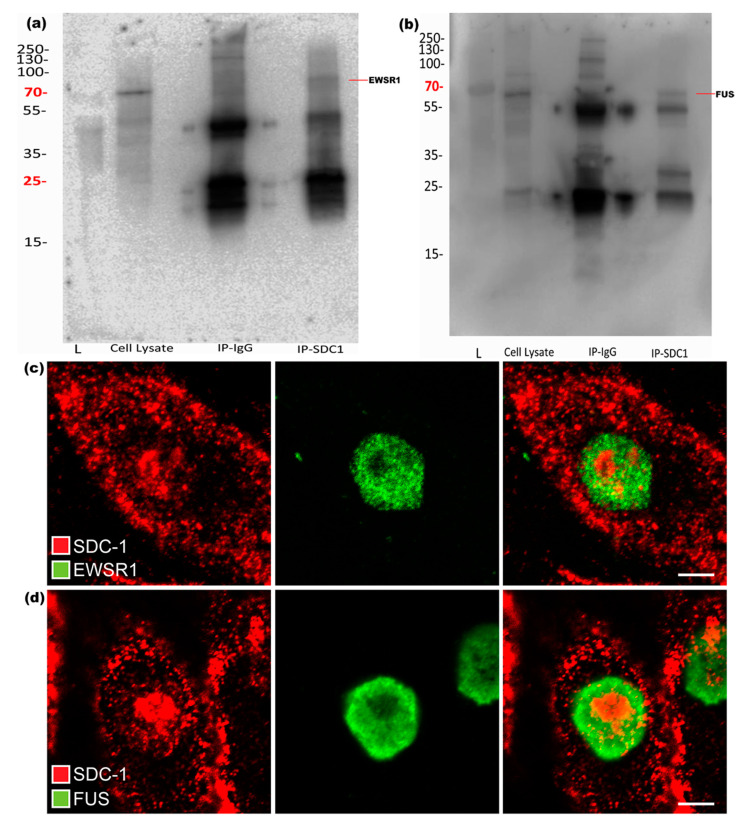
Validation of interaction between SDC1 and EWSR1 or FUS, respectively. Western blots of SDC1 immunoprecipitates. (**a**) The blot shows presence of EWSR1. (**b**) The presence of FUS in SDC1 immunoprecipitate. “L” represents ladder in both the blots. (**c**,**d**) The figure shows the presence of SDC1 (red) along with EWSR1 (green) and FUS (green) in the nucleus, respectively.

Results from IP with both used SDC1 antibodies and subsequent Western blot experiments confirmed that EWSR1 and FUS could be precipitated together with SDC1, as shown in Figure 3a,b. To further validate and show the presence of these proteins along with SDC1, immunofluorescence studies were performed. Although SDC1 and both the selected key proteins were simultaneously present in the nuclei, there was little evidence of co-localization at the selected resolution, indicating that the Co-IP pulled down large networks with a distance between the key proteins and SDC1, as shown in Figure 3c,d.

**Figure 4:**
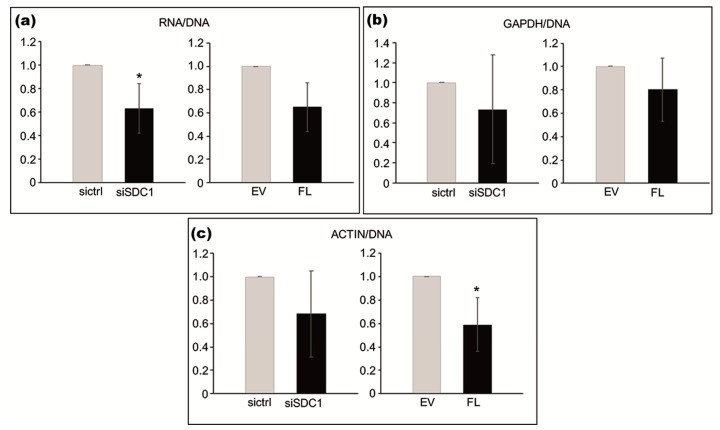
Effect of loss- and gain-of-SDC1 on cellular amounts of total RNA and specific RNA for actin, relative to DNA content. (**a**–**c**; left images) Bar graphs showing the effects of knocking down SDC1 by siRNA (siSDC1) versus control (sictrl) on total RNA (**a**), actin (**b**), and GAPDH (**c**) RNA levels. (**a**–**c**; right images) Bar graphs showing the effects of overexpressing full-length SDC1 (FL) versus an empty vector (EV) on total RNA (**a**) actin (**b**) and GAPDH (**c**) RNA levels. The results are based on three independent experiments. Asterisks indicate statistical significance from the corresponding controls (* *p* < 0.05).

Based on the identified association between SDC1 and proteins involved in RNA synthesis, we used loss- and gain-of-function experiments to study whether SDC1 levels influence cellular amounts of RNA. The total amount of RNA, normalized to DNA content, was significantly lower in SDC1 knockdown cells compared to control cells (*p* = 0.0379), as shown in Figure 4 in the left image. A tendency towards lower levels of RNA was also seen in cells after the overexpression of SDC1 (*p* = 0.0689), as shown in Figure 4a in the right image. Further analysis by qPCR was performed to study whether SDC1 levels specifically affected RNA levels of the two housekeeping genes, Actin and GAPDH. The knockdown of SDC1 had no significant effect on total RNA levels (*p* = 0.212), as shown in Figure 4b in the left image, while the overexpression of SDC1 resulted in decreased levels of Actin RNA (*p* = 0.0364), as shown in Figure 4b in the right image, compared to the controls.

## 4. Discussion

It has been known for decades that heparan sulphate (HS) [28,29,30,31] and SDC1 can be present in the nucleus of mesothelioma various cells [7], but little is known about their possible functions and nuclear interactions [7,9,32,33,34,35]. In a previous study, we demonstrated that the upregulation of SDC1 hampers cell proliferation and that the localization of SDC1 is important for the fate of tumor cells [36]. This decreased cell growth seems to mainly depend on the cytoplasmic/transmembrane domains [12]. Interestingly, proliferation also decreases when the SDC1 expression is decreased with interfering RNA [14].

The aim of this study was to obtain further insight into the role of nuclear SDC1 by characterizing its nuclear interactome in a mesothelioma cell line, which was previously used to identify the function of SDC1 in cell proliferation. As expected, nuclear proteins involved in cell proliferation were present in the SDC1 precipitate. More surprisingly, we found that SDC1 also associated with proteins involved in RNA synthesis.

During the bioinformatic analysis, two sets of pathway analyses were performed to see the overrepresented significant pathways. In the first set, all the interacting proteins, irrespective of their localizations, were selected for pathway analysis, indicating the overall involvement of SDC1 in 28 different pathways. When the analysis was limited to proteins ascribed nuclear localization only, eight significant pathways were indicated as being influenced by nuclear SDC1, as shown in Table 1.

Of these eight pathways, two seemed to be irrelevant in the present context (oocyte meiosis and herpes infection), and they were not subjected to further exploration. One of the remaining six functions influenced by nuclear SDC1 concerns cell proliferation, which is in accordance with the above described previous findings that cell proliferation decreases following transfection with full length SDC1 carrying the nuclear localization signal RMKKK, as well as when the SDC1 expression is suppressed. The present results indicate that this regulatory effect on cell proliferation is related to SDC1 when present in the nucleus.

The remaining five pathways were all associated with various aspects of RNA transcription and export, as shown in Table 1. Two pathways, mRNA surveillance and Spliceosome, have members of EJC (Exon junction complex) in common, thereby linking them together. We hypothesize that SDC1 might be regulating the binding of EJC and mRNA and further assisting in the degradation of the mRNA via different mechanisms [37]. The EJC complex is known to regulate translation, mRNA surveillance and mRNA localization [37,38,39]. In the present study, three EJC inner core factors, namely Y14, MAGOH and eIF4AIII, were found to interact with SDC1. Apart from the core factors of the EJC complex, two transiently interacting factors, Upf1 and SRm160, were also identified as interacting partners in this study. The role of SRm160 has been described elsewhere [40]. These transient factors play important roles in nonsense-mediated decay [41]. Upf1 is phosphorylated and, in turn, leads to mRNA degradation. In mRNA decay, decapping also plays an important part. Interestingly, two factors, EDC3 (enhancer of mRNA-decapping protein 3) and EDC4 (enhancer of mRNA-decapping protein 4), were also identified as interactors of SDC1. Previous studies reported that EDC3 does not affect the nonsense-mediated decay pathway [42]. Apart from decapping, EDC4 was reported to have a role in the DNA repair mechanism [43]. EDC3 and EDC4 assist in mRNA decapping and the degradation process [44,45]. The exact mechanism and role of SDC1 in the whole process is still not known and it is yet to be proved in vitro and/or in vivo. In general, the total cellular amount of RNA decreased following both the up- and downregulation of SDC1, similar to the effects on cell proliferation. A similar trend for nuclear SDC1 on RNA transcription and export was seen when quantifying RNA, coding for two housekeeping genes—GAPDH and actin. When the expression of SDC1 is quantified, related to the GAPDH as a housekeeping gene, the reduced GAPDH, seen with SDC1 silencing, will cause the underestimation of how far the SDC1 is silenced.

The nuclear compounds that interact directly or indirectly with SDC1 form large networks, including hundreds of different proteins. These networks regulate two main functions: cell proliferation and the transcription modification and transport of RNA. When the amounts of SDC1 are altered, this hampers the respective functions, regardless of if SDC1 is up- or downregulated, and it seems that the effects of SDC1 on these functions are regulatory, rather than simple stimulation or blocking. A similar “bell shaped” dose-response effect of varying the expression of SDC1 has been demonstrated with a number of genes and pathways [14]. The finding of an optimal response over a rather narrow concentration range has been shown earlier in signaling mediated by heparan sulfate or growth factors. This seems to also be valid for networks associated with nuclear proteins, including EWSR1 and FUS, which were identified as proteins co-precipitating with SDC1. Interestingly, malignant mesotheliomas were recently found to be associated with recurrent EWSR1/FUS fusions [46].

## 5. Conclusions

The present study provides new insight into the role of nuclear SDC1 in a mesothelioma cell line. Functional classification indicated that SDC1 plays a role in various pathways, ranging from cell proliferation to RNA synthesis and transport. A limitation of the study is that the results are based on SDC1 interacting proteins in a single mesothelioma cell line and, therefore, do not allow for generalization about the SDC1 nuclear interactome in other cells. Further studies are needed to elucidate the precise molecular mechanisms of nuclear SDC1 and its direct involvement in these pathways, as well as to study its possible importance in tumors, such as malignant mesothelioma.

## Figures and Tables

**Table 1:** Significant pathways where SDC1 interacts with nuclear proteins.

KEGG_ID	Pathways	*p*-value
hsa03040	Spliceosome	<0.001
hsa03010	Ribosome	<0.001
hsa03013	RNA transport	<0.001
hsa03015	mRNA surveillance pathway	<0.001
hsa05168	Herpes simplex infection	0.001
hsa03450	Non-homologous end-joining	0.002
hsa04110	Cell cycle	0.036
hsa04114	Oocyte meiosis	0.036

**Table 2:** The table represents interacting proteins of SDC1 having higher peak area values. The values were rounded off to the nearest whole number.

Protein	Function	Average Peak Area
EWSR1	Ewing sarcoma RNA Binding Protein, gene expression, cell signaling, RNA processing and transport.	1017854433
FUS	DNA/RNA-binding protein, transcription regulation, RNA splicing, RNA transport, DNA repair and damage response	605530108
SRSF3	Serine and Arginine Rich Splicing Factor 3	385181348
HNRPNK	Heterogeneous nuclear ribonucleoprotein K, pre-mRNA processing and mRNA metabolism and transport.	295093486
HNRH1	Heterogeneous nuclear ribonucleoprotein H isoform X2	187344787
DDX5	DEAD box protein 5 or RNA helicase p68, translation initiation and ribosome and spliceosome assembly.	170687719
NONO	Non-POU domain-containing octamer-binding protein, many nuclear processes and binds to both DNA and RNA	160241747
SRSF1	Serine/arginine-rich splicing factor 1 (SRSF1)	147735561
EDC4	Enhancer of mRNA-decapping protein 4.	140588443
RENT1	UPF1 RNA Helicase and ATPase, mRNA nuclear export and mRNA surveillance	132528140
TAF15	TATA-binding protein-associated factor 2N, basal transcription	128505347
MYO1C	Myosin-Ic, the nuclear isoform associates with RNA polymerase I and II and functions in transcription initiation.	113213904
DDX17	DEAD box protein 17, RNA helicases	111436913
SRSF7	Serine/arginine-rich splicing factor 7 (SRSF7)	99271832
EIF3C	Eukaryotic Translation Initiation Factor 3 Subunit C	85388787
EIF3L	Eukaryotic Translation Initiation Factor 3 Subunit L	78620983
HNRPM	Heterogeneous nuclear ribonucleoprotein M, mRNA splicing via spliceosome	77690416
ILF3	Interleukin enhancer-binding factor 3, RNA-binding protein, biogenesis of circular RNAs (circRNAs)	77171065
SRSF4	Splicing factor, arginine/serine-rich 4	44588287
THOC4	THO complex subunit 4, nuclear export of spliced and unspliced mRNA.	41230482
DHX15	Putative pre-mRNA-splicing factor ATP-dependent RNA helicase DHX15	35497317
XRCC5	Ku80 protein, DNA repair	35470341
ABCF1	ATP-binding cassette sub-family F member 1, plays a role in enhancement of protein synthesis and inflammation	15304939
EIF5B	Eukaryotic translation initiation factor 5B	10174063

## Supplementary Materials

The following are available online at https://www.mdpi.com/2218-273X/10/7/1034/s1, Figure S1: Western blot showing the presence of SDC1. Figure S2: The workflow adopted for the analysis. Figure S3: Proteins interacting with SDC1 according to the STRING database. Figure S4: The figure shows overrepresented pathways predicted using WebGestalt tool. (a) The overrepresented pathways of all the interacting proteins of SDC1. (b) The over-represented pathways of nuclear proteins interacting with SDC1. The bars are colored on the basis of FDR. Figure S5: The figure shows the link between the pathways (Ribosome, mRNA surveillance, RNA transport and Spliceosome). Table S1: The table includes all the identified proteins in the control and SDC-IP samples and the sub-cellular localization of the proteins interacting with SDC1.
