# Supplementary material for: Mapping the Interactome of the Nuclear Heparan Sulfate Proteoglycan Syndecan-1 in Mesothelioma Cells
Source: Biomolecules. 2020 Jul 11;10(7):1034. doi: 10.3390/biom10071034 (PMC7408266; doi:10.3390/biom10071034)
Supplement: Supplementary file 1 [file biomolecules-10-01034-s001.zip › biomolecules-744822-supplementary.html]

Supplementary Materials-2.pdf | Använder Box   


               

JavaScript is currently disabled in your browser. You must have JavaScript enabled to take full advantage of Box.

Växla till Box-versionen med hjälpmedelsfunktioner ›

Logga inRegistrering

1. # MS\_100720

Hämta

Namn

Uppdaterad

Storlek

FigS5.tif

Idag av Ashish Kumar Singh

138,5 MB

Fil

AS\_AD\_FS\_JS\_manuscript744822PROOFS.v6.corr.doc

Idag av Ashish Kumar Singh

23,4 MB

Fil

Supplementary Materials-2.docx

Idag av Ashish Kumar Singh

4,7 MB

Fil

FigS5.pdf

Idag av Ashish Kumar Singh

316,5 KB

Fil

Supplementary Materials-2.pdf

Idag av Ashish Kumar Singh

862,5 KB

Information

## Filegenskaper

Ägare
:   Ashish Kumar Singh

Företagsägare
:   Karolinska Institutet

Uppladdare
:   Ashish Kumar Singh

Skapad
:   10 juli 2020 11:27

Ändrad
:   10 juli 2020 11:27

Storlek
:   138,5 MB

1. Supplementary Materials-2.pdf
2. MS\_100720·Uppdaterad Idag av Ashish Kumar Singh

HämtaRegistreringLogga in

✕

Mapping the interactome of the nuclear heparan sulfate proteoglycan syndecan-1 in mesothelioma cellsAshish Kumar-Singh1,JatinShrinet2, Malgorzata MariaParniewska1, Jonas Fuxe1,Katalin Dobra1,3,\*and Anders Hjerpe1,31Division of Pathology, Department of Laboratory Medicine, Karolinska Institutet, SE-14186 Stockholm, Sweden; Ashish.Kumar.Singh@ki.se(A.K.-S.); jonas.fuxe@ki.se(J.F.); malgorzata.parniewska@ki.se(M.P.)2Department of Biological Science, FloridaState University, Tallahassee-32306, FL, USA; jatbioinfo@gmail.com3Division of Clinical Pathology/Cytology, Karolinska University Laboratory, Karolinska University Hospital, SE-14186 Stockholm, Sweden\*Correspondence: Katalin.dobra@ki.se(K.D.); anders.hjerpe@ki.se(A.H.); Tel.:+46-8-4841093

Supplementary MaterialFigure S1SDC1 antibody evaluatedin western blot.30ug of antibody gave the optimalyield of immunoprecipitated SDC1.Figure S2The workflow adopted for the analysis.

Figure S3Proteins interacting with SDC1:The interaction network was generated merging the data downloaded from STRING database and the interactors identified in thepresent study.

Figure S4The figure represents top enriched pathways. (a) Pathway analysis of all the interacting partners of SDC1. (b) Pathway analysis of nuclear localized interacting partners of SDC1. Dark blue color represents withpathways FDR ≤0.05 and light blue color represents over-represented pathways FDR>0.05.

Figure S5The figure shows link between the pathways (Ribosome, mRNA surveillance, RNA transport and Spliceosome).Two pathways namely, mRNA surveillance and Spliceosome are also shown in insat. EJC complex are highlighted.

Läser in förhandsgranskning …

Hämta fil

106%

1 / 6

### Information

Filegenskaper

Ägare
:   Ashish Kumar Singh

Uppladdare
:   Ashish Kumar Singh

Skapad
:   10 juli 2020 11:50

Ändrad
:   10 juli 2020 11:50

Storlek
:   862,5 KB

Släpp filer på den här sidan för att ladda upp dem till den här mappen.
